# Tubular aryl hydratocarbon receptor upregulates EZH2 to promote cellular senescence in cisplatin-induced acute kidney injury

**DOI:** 10.1038/s41419-022-05492-3

**Published:** 2023-01-12

**Authors:** Li Wen, Qian Ren, Fan Guo, Xiaoyan Du, Hongliu Yang, Ping Fu, Liang Ma

**Affiliations:** 1 Kidney Research Institute, Department of Nephrology, West China Hospital of Sichuan University, Chengdu, 610041 China; 2 Department of Nephrology, The Affiliated Hospital of Southwest Medical University, Luzhou, 646000 China; 3 Department of Pharmacy, West China Hospital, Chengdu, 610041 China

**Keywords:** Acute kidney injury, Aryl hydrocarbon receptor, Enhancer of zeste homolog 2, Cellular senescence., Diseases, Kidney diseases

## Abstract

Acute kidney injury (AKI) is one of the serious clinical syndromes with high morbidity and mortality. Despite substantial progress in understanding the mechanism of AKI, no effective drug is available for treatment or prevention. In this study, we identified that a ligand-activated transcription factor aryl hydrocarbon receptor (AhR) was abnormally increased in the kidneys of cisplatin-induced AKI mice or tubular epithelial TCMK-1 cells. The AhR inhibition by BAY2416964 and tubular conditional deletion both alleviated cisplatin-induced kidney dysfunction and tubular injury. Notably, inhibition of AhR could improve cellular senescence of injured kidneys, which was indicated by senescence-associated β-galactosidase (SA-β-gal) activity, biomarker p53, p21, p16 expression, and secretory-associated secretory phenotype IL-1β, IL-6 and TNFα level. Mechanistically, the abnormal AhR expression was positively correlated with the increase of a methyltransferase EZH2, and AhR inhibition suppressed the EZH2 expression in cisplatin-injured kidneys. Furthermore, the result of ChIP assay displayed that EZH2 might indirectly interact with AhR promoter region by affecting H3K27me3. The direct recruitment between H3K27me3 and AhR promoter is higher in the kidneys of control than that of cisplatin-treated mice, suggesting EZH2 reversely influenced AhR expression through weakening H3K27me3 transcriptional inhibition on AhR promoter. The present study implicated that AhR and EZH2 have mutual regulation, which further accelerated tubular senescence in cisplatin-induced AKI. Notably, the crucial role of AhR is potential to become a promising target for AKI.

## Introduction

Acute kidney injury (AKI) is a common clinical syndrome and a major health issue, which refers to a rapid decline of kidney function in a short period of time caused by a variety of factors, such as renal hypoperfusion, trauma, sepsis, and toxic drugs, etc [1–3]. Approximately 13.3 million people suffer from AKI every year [4], and 30 ~ 70% of AKI patients could develop into chronic kidney disease (CKD) or end-stage kidney disease (ESKD) [5], and about 1.7 million of the deaths are caused by AKI [6, 7]. As we known, cisplatin is an antitumor chemotherapy drug, but one-third of cancer patients receiving cisplatin chemotherapy are susceptible to AKI [8]. However, practical strategies for treating cisplatin-induced AKI are still lacking. Therefore, focusing on the mechanism of cisplatin-induced AKI is of great significance for drug discovery and the improvement in the quality of the population associated with cisplatin nephrotoxicity.

The increasing studies reported that cisplatin could stimulate oxidative stress and induce cellular senescence [9], which even may promote AKI to CKD progression [10]. Notably, oxidative stress has emerged as a major cause of cellular senescence [9] and oxidative stress-mediated cellular senescence was involved in the process of cisplatin-induced interstitial fibrosis [11, 12]. In cisplatin-induced cultured tubular epithelial cells, mitochondrial dysfunction, accompanied by excessive reactive oxygen species (ROS) production, leads to severe damage [13]. Mitochondrial dynamics improvement and cellular premature senescence repression contribute to exerting renoprotective effects [12, 14]. To date, considerable studies have focused on cisplatin nephrotoxicity, whereas very little is known about molecular mechanism by which cisplatin caused cellular senescence in the injured kidneys.

Aryl hydrocarbon receptor (AhR) is a ligand-activated transcription factor, which can change conformational and expose the nuclear transfer site after binding to its ligand, along with binding to aromatic hydrocarbon receptor nuclear transfer proteins (ARNT) to regulate the expression of target genes [15, 16]. Few efforts have play attention to AhR-related mechanisms of AKI until now. Our previous study showed that AhR activation triggered inflammation and apoptosis in rhabdomyolysis and ischemia- reperfusion (IR) induced AKI [17]. Furthermore, AhR pathway activation was reported to contribute to tubular epithelial cell senescence under anoxia or reoxygenation [18]. Dramatic AhR increase in cisplatin-induced AKI points to AhR as a critical factor inducing oxidative stress. However, whether AhR upregulation participates in cellular senescence of cisplatin-induced AKI and its potential mechanisms need to be further explored.

Thus, in the study, we aimed to explore the potential mechanism of AhR-mediated senescence and identify AhR as a potential drug target against cisplatin-induced AKI. Here, the present results indicated that the inhibition of AhR alleviated cisplatin-induced oxidative stress and cell senescence, further protected against kidney dysfunction and tubular injury, which may be closely related to the reduction of EZH2, caused by AhR inhibition.

## Results

### Abnormal expression of tubular AhR aggravated cisplatin-induced AKI

**Fig. 1:**
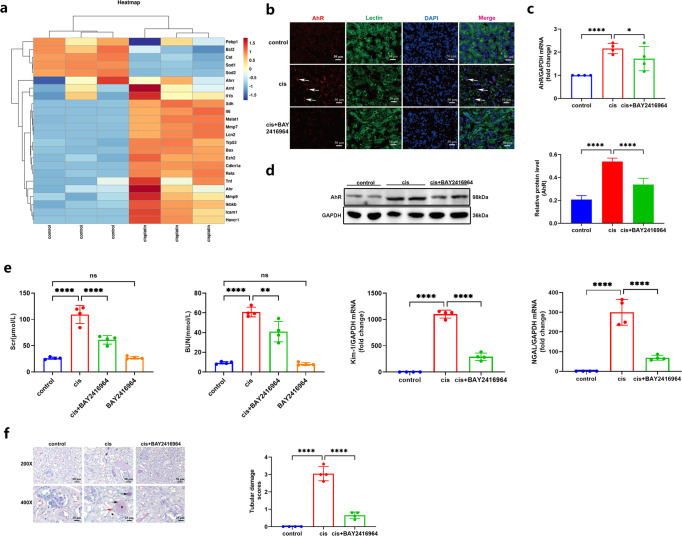
AhR antagonist BAY2416964 alleviated tubular injury in cisplatin-induced AKI mice. **a** The cluster heatmap was conducted to present differentially expressed genes in the GSE106993 dataset (*n* = 3). **b** Representative micrographs of AhR (red) with tubule marker Lectin (green) by immunofluorescence staining of kidney tissues (×200). **c**, **d** The mRNA and protein levels of AhR, and the relative protein level of AhR was quantified by densitometry and normalized with GAPDH (*n* = 3); All western blot protein samples were taken from two random mice in a different group, and the experiments were repeated three times. **e** The biochemical levels and mRNA expression of Kim-1 and NGAL (*n* = 4); **f** Representative PAS staining sections of kidney tissues were magnified ×200 and ×400, and tubular damage scores were calculated. Data are represented as means ± SDs (*n* = 4). cis, cisplatin. ****P < 0.0001, **P < 0.01, *P < 0.05, ^ns^*P* > 0.05.

The characteristics of AhR in kidneys by public data of single-cell sequencing were shown in Supplementary Fig. 1. There was a highly increase of AhR expression across proximal tubule clusters after renal ischemia reperfusion injury at 4 h and we could propose that the expression of AhR in tubular epithelial cells is up-regulated in ischemia reperfusion-induced transient kidney injury. Thus, based on the above single-cell data, we turn to consult the Gene Expression Omnibus database (No. GSE106993) and checked the RNA-sequencing data about cisplatin-stimulated mice [19]. A clustered heatmap was shown in Fig. 1a, the level of AhR mRNA was significantly upregulated in the kidneys of cisplatin-induced mice compared with that of control. Subsequently, we used the immunofluorescence staining to detect the location and expression of AhR protein in kidneys of cisplatin-induced mice. In the inured kidneys of mice, the fluorescence intensity of AhR was enhanced in the renal tubule nucleus, indicating the upregulation and activation of AhR in tubules (Fig. 1b), which the expression of AhR was markedly reduced by an antagonist BAY2416964 treatment. Consistently, the levels of AhR mRNA and protein were abnormally increased after cisplatin administration, whereas BAY2416964 treatment substantially decreased the corresponding expression (Fig. 1c, d).

Importantly, the inhibition of AhR by BAY2416964 improved the Scr, BUN levels of kidney dysfunction, and inhibited mRNA expression of injury biomarker havcr1 (kidney injury molecule 1, Kim-1) and Lcn2 (neutrophil gelatinase-associated lipocalin, NGAL) in cisplatin-induced mice (Fig. 1e). BAY2416964 treatment alone did not affected these corresponding indicators (Fig. 1e). Meanwhile, the result of PAS staining displayed that AhR repression improved kidney pathological damages in the cisplatin-induced mice, which was characterized by renal tubular dilation, loss of brush border, cast formation, and tubular epithelial cell apoptosis or necrosis (Fig. 1f).

**Fig. 2:**
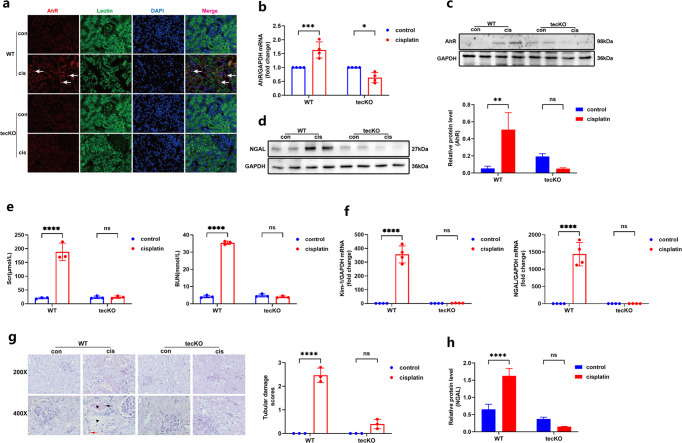
Tubular epithelial cell-specific AhR deletion improved kidney injury in cisplatin-induced mice. **a** Representative micrographs of AhR (red) and tubule marker Lection (green) by immunofluorescence staining in kidneys of cisplatin-induced WT or AhR tecKO mice (×200). **b** The AhR mRNA level by RT-qPCR (*n* = 4). **c** The AhR protein level by western blot and the relative protein level was quantified by densitometry and normalized with GAPDH (*n* = 3). **d**, **h** NGAL expression and the relative protein level was quantified by densitometry and normalized with GAPDH (*n* = 3); All western blot analysis was performed in two randomized mice from each group, and the experiments were repeated in triplicate. **e** Scr and BUN levels in cisplatin-induced WT or AhR tecKO mice. **f** The Kim-1, NGAL mRNA levels by RT-qPCR (*n* = 4). **g** Representative PAS staining sections (×200 and ×400) and tubular damage scores (*n* = 3). Data are represented as means ± SDs. cis, cisplatin. ****P < 0.0001, **P < 0.01, *P < 0.05, ^ns^*P* > 0.05.

Consistent with the abovementioned results, the role of AhR was explored in tubular epithelial cell-specific deletion (tecKO) mice. The upregulation and activation of AhR were observed in WT cisplatin mice, but not in cisplatin tecKO mice (Fig. 2a–c). Moreover, kidney dysfunction as well as the mRNA and protein expression of Kim-1, NGAL were dramatically elevated in cisplatin-injected WT mice. As expected, conditional knockout of AhR played a positive renal protective effect, and the increase of corresponding indicators was not found in cisplatin-induced tecKO mice (Fig. 2d–f, h). Importantly, conditional knockout of AhR could alleviate kidney pathological damages in cisplatin-induced AKI (Fig. 2g). Taken together, these data indicated that the inhibition of AhR protected against tubular injury of cisplatin-induced mice.

### AhR promotes cellular senescence in kidneys of cisplatin-induced mice

**Fig. 3:**
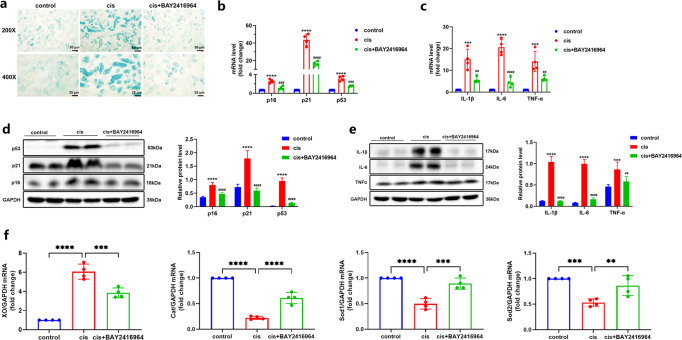
AhR inhibition by BAY2416964 suppressed senescence phenotype and oxidative stress in kidneys of cisplatin-induced mice. **a** Representative senescence photographs of SA-β-Gal staining in cisplatin-induced kidneys (magnification ×200 and ×400); Blue: SA-β-gal positive region. **b** The mRNA levels of p16, p21, and p53 in different groups of mice (*n* = 4). **c** The mRNA levels of IL-1β, IL-6 and TNFα (*n* = 4). **d** Protein expression of p16, p21 and p53 were quantified by densitometry and normalized with GAPDH. **e** Protein expression of IL-1β, IL-6, and TNFα were quantified by densitometry and normalized with GAPDH; ****P < 0.0001, ***P < 0.001 compared with the control group; ^####^*P* < 0.0001, ^###^*P* < 0.001, ^##^*P* < 0.01 compared with cisplatin group. All western blot analysis was performed in two randomized mice from each group, and the experiments were repeated in triplicate. **f** The mRNA expression of XO, Cat, Sod1, and Sod2 (*n* = 4); Data are represented as means ± SDs. cis, cisplatin. ****P < 0.0001, ***P < 0.001, **p < 0.01.

Generally, the injured kidney could become a normal or near-normal kidney through the following tissue repair mechanisms, such as inflammatory infiltrate resolution, tubular proliferation, and epithelial repair or regeneration [20]. However, maladaptive repair causes cell cycle arrest in the G2/M phase and releases senescence-associated secretory phenotypes (SASP), which are critical risk factors of CKD progression after AKI [20]. To explore whether AhR-induced tubular injury is related to cellular senescence, we detected the expression of senescence-associated β-galactosidase (SA-β-gal) in the kidneys of cisplatin-induced mice and the SA-β-gal positive area was significantly increased in the cisplatin-induced renal tubular cells, which was inhibited by AhR suppression by BAY2416964 (Fig. 3a). Meanwhile, we observed an obvious upregulation of senescence-associated genes (SAGs: p16, p21 and p53) and SASP (IL-1β, IL-6, TNF-α) in the kidneys of cisplatin mice, and further BAY2416964 treatment repressed the expression of the corresponding SAGs and SASPs (Fig. 3b–e). It has been reported that AhR activation in the heart generated excessive ROS [21], which may be responsible for cellular senescence [22]. Thus, oxidative stress was evaluated in the kidneys of cisplatin-induced mice. Consistent with the previous results [23], xanthine oxidase (XO) level was increased, and anti-oxidant enzymes (Cat, Sod1, Sod2) levels were decreased in kidneys of cisplatin mice (Fig. 3f). These results supported a conclusion in which AhR activation-induced oxidative stress by stimulating oxidase and repressing antioxidant enzymes. All above, AhR activation in cisplatin-induced AKI mice may promote senescence phenotype, which was mediated by oxidative stress, whereas the specific mechanisms between them needs to be further explored.

**Fig. 4:**
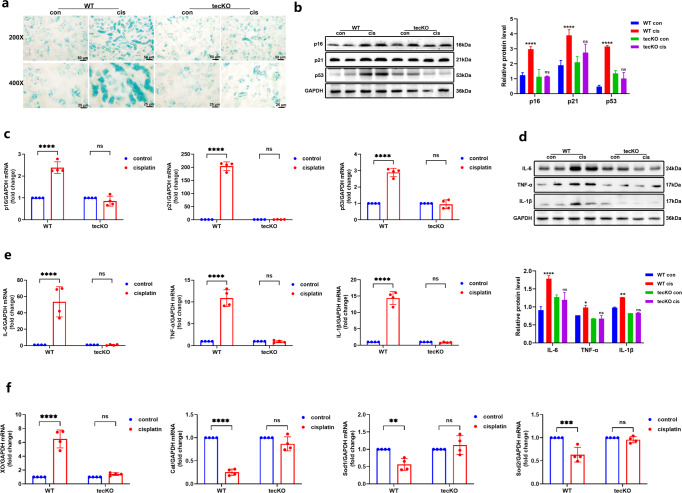
Tubular AhR deletion improved cellular senescence and oxidative stress in cisplatin-induced AKI mice. **a** Representative images of SA-β-Gal staining in cisplatin-induced WT and AhR tecKO mice (magnification ×200 and ×400). Blue: SA-β-Gal positive region. **b** Protein expression of SAGs (p16, p21, p53) and **d** SASPs (IL-1β, IL-6, TNF-α) shown by western blot in WT and AhR tecKO mice with or without cisplatin administration, and the relative protein level was quantified by densitometry and normalized with GAPDH (*n* = 3); ****P < 0.0001, **P < 0.01, *P < 0.05 compared with WT control group; ^ns^*P* > 0.05 compared with tecKO control group. All western blot protein samples were taken from two random mice in different group, and the experiments were repeated three times. **c** Transcript expression of SAGs (p16, p21, p53) and **e** SASPs (IL-1β, IL-6, TNF-α) in WT and AhR tecKO mice with or without cisplatin administration (*n* = 4). **f** Oxidant (XO) and anti-oxidant (Cat, Sod1, Sod2) expression, measured by RT-qPCR in WT and AhR tecKO mice with or without cisplatin administration (*n* = 4). Data are represented as means ± SDs. cis, cisplatin. ****P < 0.0001, ***P < 0.001, **p < 0.01, ^ns^*P* > 0.05.

Consistently, the lower SA-β-gal positive area in kidneys of cisplatin-induced AhR tecKO mice was detected compared with that of WT mice (Fig. 4a). Furthermore, the expression of SAGs (p16, p21 and p53) and SASPs (IL-1β, IL-6, TNF-α) were substantially inhibited in kidneys of cisplatin-induced AhR specific deficient mice (Fig. 4b–e). Similarly, the up-regulation of oxidase and the down-regulation of antioxidant enzymes in injured kidneys of WT mice were observed, which were not present in cisplatin-indued AhR tecKO mice. The results could confirm that AhR activation participates in regulating oxidative stress and cellular senescence in injured kidneys.

### Knockdown of AhR alleviates senescence and oxidative stress in cisplatin-stimulated tubular epithelial TCMK-1 cells

**Fig. 5:**
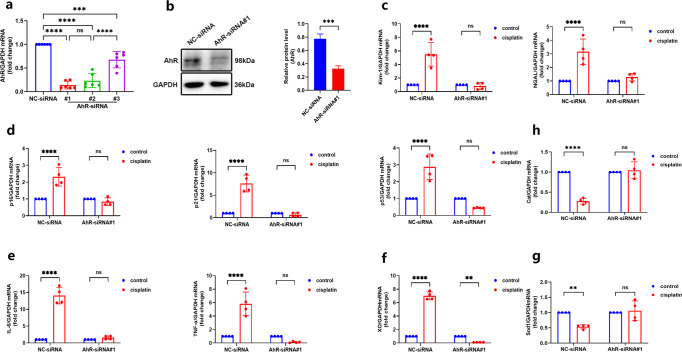
AhR knockdown attenuated cellular injury and senescence as well as represses oxidative stress in cisplatin-stimulated tubular epithelial TCMK-1 cells. **a** Screening for suitable AhR-siRNA. **b** AhR protein level in TCMK-1 cells, and the relative protein level was quantified by densitometry and normalized with GAPDH (*n* = 3). All western blot experiments were repeated in triplicate. **c** Transcript expression of Kim-1 and NGAL in TCMK-1 cells with or without cisplatin stimulation (*n* = 4). **d** Transcript expression of SAGs (p16, p21, p53) and **e** SASPs (IL-1β, IL-6, TNF-α) in TCMK-1 cells with or without cisplatin stimulation (*n* = 4). **f** Oxidant (XO) and **g**, **h** anti-oxidant (Cat, Sod1) expression, measured by RT-qPCR in TCMK-1 cells with or without cisplatin stimulation (*n* = 4). Data are represented as means ± SDs. cis, cisplatin. ****P < 0.0001, ***P < 0.001, **p < 0.01, ^ns^*P* > 0.05.

Next, we carried out a siRNA transfection test to see if AhR knockdown could exert anti-senescent and anti-oxidant stress effect in vitro. The best silencing efficacy of AhR-siRNA#1 from the three transfection sequences was selected, where the mRNA and protein levels of AhR-siRNA#1 were markedly reduced (Fig. 5a, b). After cisplatin stimulation, the mRNA levels of Kim-1 and NGAL were up-regulated, while AhR knockdown improved cell injury (Fig. 5c). Consistent with the in vivo result, in cisplatin-induced TCMK-1 cells, AhR knockdown attenuated cisplatin-stimulated cellular senescence (Fig. 5d, e), as evidenced by the reduction of SAGs (p16, p21 and p53) and SASPs (IL-6, TNF-α). Compared with negative control group, cisplatin stimulation up-regulated XO and down-regulated Sod1, Cat, while AhR knockdown reversed the expression of oxidative stress-related factors and suppressed oxidative stress response (Fig. 5f–h).

### AhR regulated EZH2 expression in cisplatin-injured kidneys and TCMK-1 cells

**Fig. 6:**
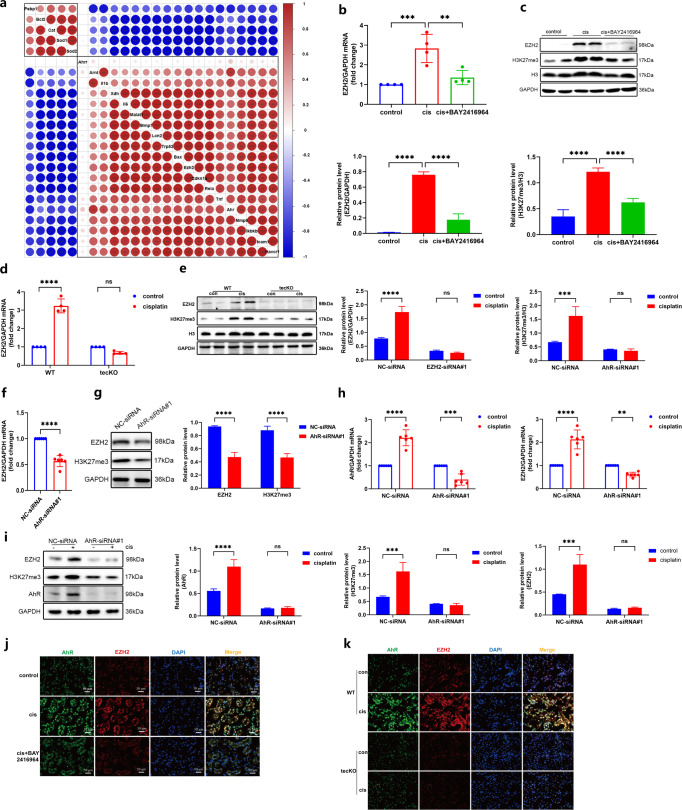
AhR triggered EZH2 and H3K27me3 expression after cisplatin stimulation. **a** Correlation in differentially expressed genes were displayed by the correlation heatmap. **b** The mRNA level of EZH2 in different groups of mice (*n* = 4). **c** Protein expression of EZH2 and H3K27me3 were shown, and gray densitometry were quantified and normalized with GAPDH (*n* = 3). **d** The mRNA level of EZH2 in WT and AhR tecKO mice with or without cisplatin administration (*n* = 4). **e** Protein expression of EZH2 and H3K27me3 were shown in kidneys of WT and AhR tecKO mice with or without cisplatin administration, and the relative protein level was quantified by densitometry and normalized with GAPDH or Histone 3 (*n* = 3). **f** Transcript expression (*n* = 6) and **g** protein level (*n* = 3) of EZH2 in TCMK-1 cells. **h** Transcript expression (*n* = 6) and **i** protein level (*n* = 3) of AhR and EZH2 in negative control (NC)-siRNA and AhR-siRNA#1 TCMK-1 cells with or without cisplatin stimulation. All western blot experiments were repeated in triplicate. Data are represented as means ± SDs. cis, cisplatin. ****P < 0.0001, ***P < 0.001, **p < 0.01, ^ns^*P* > 0.05. **j** Representative micrographs of AhR (green**)** with EZH2 (red) in tubular cell nucleus by immunofluorescence staining in kidneys of cisplatin-induced C57BL/6 J mice (×200). **k** Representative micrographs of AhR (green) and EZH2 (red) in tubular cell nucleus by immunofluorescence staining in kidneys of cisplatin-induced WT or AhR tecKO mice (×200).

Previously, we demonstrated that inhibition of EZH2 could reduce cisplatin-induced inflammation and improve renal injury [24, 25]. In pancreatic cancer cells, the activation of AhR/EZH2 signaling axis causes epigenetic alteration [26]. Therefore, we explored the effect of AhR on the regulation of EZH2 in the kidneys of cisplatin-induced mice. We made a correlation heatmap of our previous RNA-sequencing data and confirmed that the mRNA level of AhR was highly related to EZH2 (Fig. 6a). As expected, the expression of EZH2, a transcription inhibition histone H3K27me3 (Fig. 6b, c), together with AhR expression (Fig. 1b–d), were dramatically elevated in the kidneys of cisplatin-induced mice. Notably, both the transcription and translation level of EZH2 were synchronously suppressed by AhR inhibition with BAY2416964 (Fig. 6b, c), indicating that the expression of EZH2 is potentially regulated by AhR. Next, we used AhR tecKO mice to further verify the relationship between AhR and EZH2. The upregulation of EZH2 and H3K27me3 were reversed by AhR deficiency after cisplatin administration (Fig. 6d, e). In addition, as shown in Fig. 5a, b and Fig. 6f, g, AhR knockdown simultaneously repressed the mRNA and protein expression of AhR and EZH2. Moreover, the expression of AhR and EZH2 were enhanced after cisplatin stimulation in TCMK-1 cells, whereas this upregulation was substantially reversed by AhR knockdown (Fig. 6h, i). Additionally, the results of fluorescence co-staining also showed that both AhR and EZH2 could be co-expressed in renal tubule nuclei of cisplatin-induced C57BL/6 J mice (Fig. 6j) or AhR conditional knockout WT mice (Fig. 6k), which the fluorescence intensity of EZH2 varied with that of AhR. These results demonstrated that AhR upregulated the expression of EZH2, which may be positively correlated with tubular senescence in cisplatin-induced AKI mice.

### AhR and EZH2 exerts physiological effects through reciprocally regulation

**Fig. 7:**
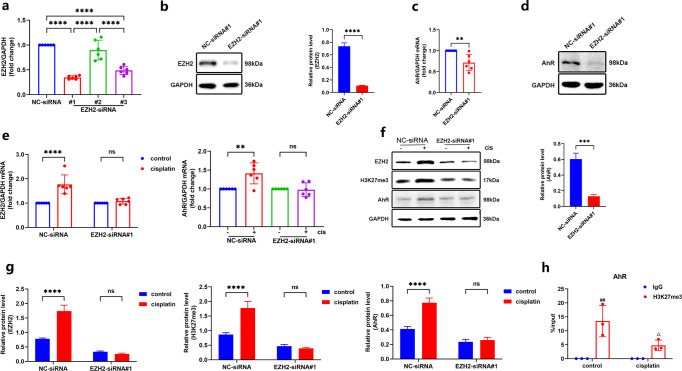
EZH2 reversely regulated AhR expression in cisplatin-stimulated tubular epithelial TCMK-1 cells. **a** Screening for suitable EZH2-siRNA through mRNA expression level. **b** EZH2 protein in negative control (NC)-siRNA and EZH2-siRNA#1 TCMK-1 cells, and the relative protein was quantified by densitometry and normalized with GAPDH (*n* = 3). **c** Transcript expression (*n* = 6) and **d** protein level (*n* = 3) of AhR in NC-siRNA and EZH2-siRNA#1 TCMK-1 cells. **e** Transcript expression (*n* = 6) and **f** protein level (*n* = 3) of EZH2 and AhR in NC-siRNA and EZH2-siRNA#1 TCMK-1 cells with or without cisplatin stimulation, **g** The relative protein level of them were quantified by densitometry and normalized with GAPDH (*n* = 3), all western blot experiments were repeated in triplicate, ****P < 0.0001, ***P < 0.001, **p < 0.01, ^ns^*P* > 0.05. **h** The kidneys from control and cisplatin-induced mice were performed to ChIP-qPCR using immunoglobulin G (IgG) and the H3K27me3 antibody (*n* = 3). Data are represented as means ± SDs. ^##^*P* < 0.01 compared with the control group IgG antibody. ^△^*P* < 0.05 compared with the cisplatin group H3K27me3 antibody. cis, cisplatin.

As found in the abovementioned results, AhR regulated the expression of EZH2 in injured kidneys. However, as an essential epigenetic regulatory enzyme, EZH2 may play a transcriptional regulation role by affecting H3K27me3. Therefore, to explore whether EZH2 reversely regulates AhR, we used EZH2 siRNA to detect the expression of AhR in vitro. The best silencing efficiency of EZH2-siRNA#1 to transfect TCMK-1 cells was selected, which the mRNA and protein level of EZH2 were dramatically reduced (Fig. 7a, b). If AhR only regulates the expression of EZH2 unidirectionally, EZH2 knockdown does not affect AhR expression. Actually, the expression of AhR was synchronously inhibited in cisplatin-triggered TCMK-1 cells by EZH2 knockdown (Fig. 7c, d), indicating that EZH2 silencing could repress the expression of AhR. Moreover, the expression of EZH2 and AhR in cisplatin-treated TCMK-1 cells was found to be markedly higher than that of the untreated group, while their expression was no longer increased by EZH2 silencing (Fig. 7e–g). These data highlighted that EZH2 reversely regulated AhR expression.

Nevertheless, how does EZH2 affect AhR expression by an epigenetic regulation? In order to address this question, we used a ChIP assay to detect the enrichment between EZH2/H3K27me3 and AhR gene promoters in vivo. There are significant overlap peaks between AhR and EZH2 promoters in non-renal cells based on the ChIP-Atlas database. Hence, we designed AhR promoter primers on the basis of the overlapping peaks, and used the ChIP-qPCR assay to detect the enrichment between EZH2 and AhR gene promoters. No enrichment between EZH2 and AhR gene promoter regions was found. AhR-binding peaks significantly overlapped with H3K27me3 in kidneys on the genome level. The ChIP-qPCR assay confirmed that H3K27me3 was bound to AhR promoter regions in kidneys of control mice, while cisplatin stimulation reduced this enrichment (Fig. 7h). These results indicated that the inhibition of H3K27me3 on AhR gene promoters was weakened, and the expression of AhR was up-regulated in cisplatin-induced AKI mice.

### Effect of EZH2 inhibition on AhR-mediated tubular epithelial cell senescence

**Fig. 8:**
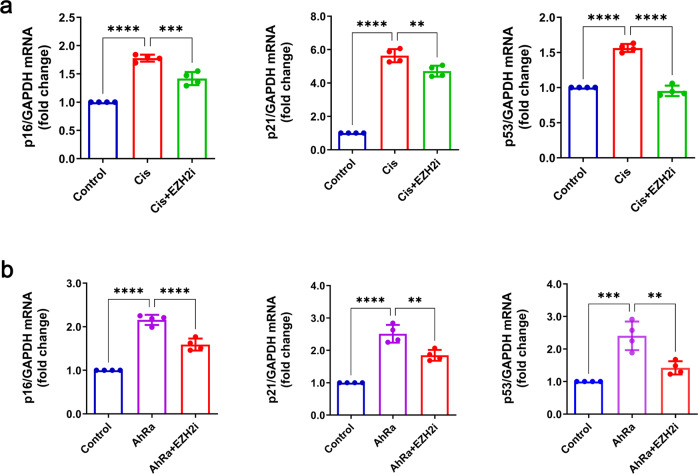
Effects of EZH2 inhibition on AhR agonism-mediated tubular epithelial TCMK-1 cell senescence. **a** The mRNA level of senescence related p16, p21, and p53 in cisplatin-induced AKI with or without EZH2 inhibitor zld1039 (EZH2i, *n* = 4). **b** The mRNA level of senescence related p16, p21 and p53 in AhR activation (AhRa) by FICZ with zld1039 (*n* = 4). Data are represented as means ± SDs. cis, cisplatin. ****P < 0.0001, ***P < 0.001, **p < 0.01, *p < 0.05.

As previously mentioned, AhR regulated cellular senescence and affected EZH2 expression in injured kidneys. However, whether EZH2 participates in the process of AhR-mediated senescence is unknown. To solve the problem, we assessed anti-senescent effect of EZH2 inhibitor zld1039 with AhR agonist FICZ in TCMK-1 cells [24, 27]. Cisplatin triggered the upregulation of SAGs (p16, p21, p53), and EZH2 inhibitor zld1039 repressed senescent mRNA level (Fig. 8a). Furthermore, whether AhR affected senescence through the direct or indirect regulation of EZH2 remain unclear. Here, we used to FICZ agonist activate AhR with the inhibition of EZH2 by zld1039 in TCMK-cells. Consequently, the senescent mRNA level of p16, p21 and p53 were increased following AhR agonist, while EZH2 inhibitor suppressed their corresponding expression (Fig. 8b). These results indicated that the upregulation of EZH2 is necessary for AhR to accelerate cisplatin-induced senescence.

## Discussion

Illustrating the potential mechanism of AhR-mediated cellular senescence is of great significance in identifying the cisplatin-induced AKI therapeutic targets. In this study, a novel finding was proposed that AhR-associated cellular senescence was involved in cisplatin-induced AKI. We found that the abnormal expression of AhR was positively associated with cisplatin-induced senescence. Inhibition of AhR by BAY2416964 or tubule-specific gene deletion significantly suppressed cellular senescence and alleviated cisplatin-induced kidney injury. Notably, further studies indicated that EZH2, a histone methyltransferase, was a pivotal factor in AhR-mediated cellular senescence in kidney injury.

Cellular senescence is a proven process of AKI maladaptive repair and kidney fibrosis [10]. A previous study has demonstrated that repeated low-dose cisplatin led to cell cycle arrest at the G2/M phase and cellular senescence, which indicated cellular senescence played a role in cisplatin-induced kidney injury [28]. Here, an increase of β-galactosidase activity, a known characteristic of senescent cells, SAGs (p16, p21, p53) and SASPs (IL-1β, IL-6, TNF-α) were also observed in the kidneys of cisplatin group compared with that of control mice. These results are consistent with those of Li et al. who confirmed that cisplatin-induced cellular senescence in tubular epithelium, could accelerate the progression of renal fibrosis [9].

AhR is an essential ligand-activated transcription factor [29]. A distinct time-dependent and tissue-specific AhR activation is displayed in different mouse models of kidney diseases [30]. Growing evidence presented that AhR activation by uremic toxins, like indoxyl sulfate, plays a harmful role in CKD progression [31, 32]. Whereas, the role of AhR in AKI remains controversial [17, 18]. Tao et al. thought that AhR activation alleviated renal injury in rhabdomyolysis and IR-induced mice by inhibiting inflammation and apoptosis [17]. Eleftheriadis et al. demonstrated that AhR pathway activation enhanced DNA damage response and promoted primary proximal renal tubular epithelial cells senescence, eventually leading to IR-induced kidney injury [18]. Therefore, the contradictory effect of AhR was explored in our study. Here, our results revealed that AhR activation accelerated the progression of kidney injury through a cellular senescence-related mechanism in a cisplatin-induced AKI mice. The AhR inhibition by BAY2416964 or tubule-specific gene deletion repressed cisplatin-induced cellular senescence, which implied that AhR might be one of the causative mechanisms of cisplatin-associated cellular senescence, and inhibition of AhR may be a promising therapeutic strategy against AKI.

Although the relationship between AhR and tubular senescence was observed in our study, we cannot clear the possible mechanisms of AhR-mediated tubular senescence in kidneys of cisplatin-induced mice. EZH2, a catalytic subunit of polycomb repressive complex 2 (PRC2), is an H3K27 histone methyltransferase [33]. Another point noticed is that H3K27me3 is mainly responsible for silencing genes, so it usually acts as a transcriptional suppressor [34]. It has been reported that EZH2 played a significant role in multiple tumor progression by affecting cellular senescence [35, 36]. In particular matter-induced skin keratinocytes, skin senescence depended on AhR-induced ROS as well as the decrease in EZH2 and H3K27me3 [37]. Meanwhile, AhR activation could enhance the EZH2 activity and increase its epigenetic silencing activity, which is a risk factor for environmental toxicant-associated pancreatitis and pancreatic cancer [26]. Not only that, EZH2 binds to the AhR promoter to repress the expression of AhR gene [38]. From this, we thought that EZH2 might play a role in AhR-mediated cellular senescence. Herein, we firstly investigated the correlation between AhR and EZH2. As expected, our results revealed that the mRNA level of AhR and EZH2 were significantly correlated in kidney RNA-sequencing data of cisplatin-stimulated mice. Cisplatin stimulation concurrently up-regulated AhR and EZH2 expression, and AhR inhibitor BAY2416964 or tubule specific AhR deficiency suppressed the expression of EZH2. In vitro, the expression of AhR and EZH2 were increased in cisplatin-treated TCMK-1 cells. The AhR knockdown reversed the elevation of AhR as well as EZH2, suggesting that EZH2 may be a potential mediator in AhR-mediated cellular senescence. Furthermore, we found that EZH2 inhibitor zld1039 indeed improved cisplatin-induced cellular senescence. Activating AhR by agonist FICZ and inhibiting EZH2 by zld1039 repressed the mRNA level of senescent p16, p21 and p53 in cisplatin-stimulated TCMK-1 cells, suggesting that EZH2 is necessary for AhR to accelerate cisplatin-induced cellular senescence.

Importantly, considering the crucial role of EZH2 in epigenetic regulation and combining with the previous results of Ko et al. [38], we further explored the influence of EZH2 knockdown on the expression of AhR. Surprisingly, EZH2 silencing reversely repressed the upregulation of AhR in cisplatin-treated TCMK-1 cells. To illustrate how EZH2 regulates AhR, we used a ChIP assay to examine the enrichment between EZH2 or H3K27me3 and the AhR promoter regions. Consequently, H3K27me3 is responsible for exerting transcriptional inhibition effect in kidneys of control mice, because of the rich enrichment between H3K27me3 and AhR promoter region, which repressed the expression of AhR. Nevertheless, the weaken enrichment of them has been described in kidneys of cisplatin mice, indicating that the rare enrichment of H3K27me3 and AhR promoter might cause cisplatin-induced AhR expression. These results identify that EZH2 also is one of the positive regulators of AhR expression by affecting the enrichment between H3K27me3 and AhR promoter.

In conclusion, our finding demonstrated that AhR was abnormally expressed in kidneys of cisplatin-induced mice and AhR inhibition alleviated cisplatin-induced cellular senescence and tubular injury against AKI. Mechanistically, our study indicated that AhR and EZH2 have mutual regulation, which accelerated tubular senescence in cisplatin-induced AKI. Notably, the crucial role of AhR is the potential to become a promising target for AKI.

## Material and methods

### Agents and antibodies

Cisplatin and BAY 2416964 were purchased from Synguider (Chengdu, China) and Selleck Chemicals (America), zld1039 was presented by the State Key of Laboratory of Biotherapy, Sichuan University (Sichuan, China), and FICZ was purchased from Selleck Chemicals (America), respectively. All primary antibodies were displayed in Supplementary Table 1.

### Animal experiments

Animal experimental procedures were licensed and permitted by the Animal Care and Use Ethics Committee of Sichuan University (2020205 A). Male C57BL/6 J mice were clarified previously [24]. C57BL/6 J mice were administered cisplatin (20 mg/kg) with or without BAY 2416964 (20 mg/kg) by intraperitoneal injection. Tubule specific AhR knockout mice were obtained from the GemPharmatech Co.,Ltd. (Jiangsu, China). AhR tecKO mice were injected cisplatin intraperitoneally to induce AKI.

### Cell culture and treatment

Mouse renal tubular epithelial cells (TCMK-1) were purchased from ATCC agency Shanghai Limai Biological Engineering Co., Ltd (Shanghai, China) and were cultured in MEM medium (G4550-500ML, Sercicebio) containing 10% fetal bovine serum (FBS) (SH30084.03, Hyclone) at 37 °C under 5% CO_2_-95% air environment. TCMK-1 cells were starved in 0.5% FBS medium for 6 h and then treated with 10 μg/ml cisplatin for another 24 h. The siRNAs were used to knock down AhR and EZH2. The transfection procedure is detailed in the riboFECT™ CP transfection kit instruction. AhR-siRNA, EZH2-siRNA and negative control (NC) siRNA were designed and synthesized by GenePharma (Shanghai, China). The detailed transfection sequences information of them is provided in Supplementary Table 2.

### Public single-cell RNA-seq analysis

The single-cell RNA sequencing database from http://humphreyslab.com/SingleCell/ was used for single-cell data analysis. According to the database, we checked the single-cell RNA sequencing data about AhR in the healthy adult humans, healthy mice, and ischemia-reperfusion injury mouse kidneys.

### Renal function

An automatic biochemical analyzer (Mindray BS-240) was taken to assess Scr and BUN. We defined that the cisplatin-induced AKI mouse model was successfully established, when the Scr value in the cisplatin group was higher than twice that in the control group.

### Pathological examination

The kidney tissues were fixed, embedded, and sectioned for Periodic Acid-Schiff (PAS) staining. Renal tubular damage semi-quantitatively scores were used to assess the pathological injury. The specific score standards and detailed rules have been shown in previous study [24].

### Immunofluorescence staining

Paraffin kidney tissue sections were firstly deparaffinized and dehydrated. Then, using the microwave method repairs the antigen. After antigen retrieval, the sections were sealed with 1× horse serum containing 0.3% Triton (Sigma, America) for 1 h at 37 °C. And then, they were incubated with primary antibody (the concentration is determined according to the instructions) overnight at 4 °C. Washing the sections and incubating the corresponding secondary antibody and lectin (1:400 dilution) for 1 h at room temperature. Then, the sections were rewashed. DAPI (D8200; Solarbio) was used to stain nuclei for 5 min. Finally, 50% glycerin was used to seal the sections. Photographs were collected from ZEN 2012 microscopy software [39].

### Senescence β-galactosidase staining

The senescence β-galactosidase staining kit (Cell Signaling Technology) was used to detect β-galactosidase activity, a known characteristic of senescent cells. For SA β-Gal staining of frozen renal tissues, frozen sections were fixed with 1× fixative solution for 10–15 min at room temperature. Washing the sections with 1×PBS. Then, added the β-galactosidase staining solution to the sections and incubated them at 37 °C overnight in a dry incubator (no CO_2_). The senescent cells showed blue color under a microscope.

### ChIP assay

Proteins and DNA interaction was evaluated by ChIP-qPCR using the ChIP assay kit (Millipore, MA, USA). The experiment protocols were according to the manufacturer’s instructions. The antibodies used for the ChIP assay were as follows: anti-H3k27me3 (Cell Signaling Technology) and control IgG (Millipore). The primers used for ChIP were as follows: AhR-F 5’-GTCAACGACATTTGCGTCCT-3’, AhR-R 5’-TCCCCTTAAGAATTTCAACTGTCC-3’. The calculation formula for enrichment efficiency was elaborated on previously [24].

### Western blot analysis

Western blotting was carried out as described earlier [24]. Densitometry analysis was evaluated by using ImageJ 6.0 software (National Institutes of Health, Bethesda, MD, USA). Gray density was normalized using internal reference proteins GAPDH or Histone 3. To ensure the repeatability of the experiment, all immunoblot bands were repeated three times.

### Quantitative real-time PCR

Total RNA separation and purification steps and RT-qPCR protocols were displayed as previously shown [24]. The corresponding gene primers were listed in Table S2. The 2^−ΔΔCt^ method was used to calculate the relative gene quantities, and GAPDH was used as the internal reference gene.

### Statistical analysis

All quantitative data were presented as mean ± standard deviation (SD). Statistical difference comparisons between the two groups were performed using the T-test. Comparisons between three or multiple groups were performed using a one-way analysis of variance (Tukey’s test). Prism 9.0 (GraphPad Software, San Diego, CA, USA) was used to draw statistical graphs, and *P*-value less than 0.05 was considered statistically significant.

## Supplementary information

Supplemental Material

Original Data File

## Data Availability

All data supporting this research has been included in this manuscript and its supplementary information files. The RNA-sequencing data for this study are available in the Gene Expression Omnibus database under accession number GSE106993. Additionally, the single-cell RNA sequencing data are from http://humphreyslab.com/SingleCell/.
