# Supplementary material for: Tubular aryl hydratocarbon receptor upregulates EZH2 to promote cellular senescence in cisplatin-induced acute kidney injury
Source: Cell Death Dis. 2023 Jan 12;14(1):18. doi: 10.1038/s41419-022-05492-3 (PMC9837170; doi:10.1038/s41419-022-05492-3)
Supplement: Supplementary file 1 — Supplemental Material [file 41419_2022_5492_MOESM1_ESM.docx]

**Supplementary table and figure legends**

**Supplementary Figure 1. The expression and characteristics of AhR in normal or abnormal kidney single cells.** All open data were from single-cell RNA sequencing database (<http://humphreyslab.com/SingleCell/>). **(a)** Collecting 4259 epithelia cells from the healthy adult human kidney, tSNE analysis presented that AhR was a few scattered within the PT cells. PT, proximal tubule; LH, loop of Henle; CD: PC, collecting duct-principal cells; DT, distal tubule; P, podocyte; CD: IC, collecting duct-intercalated cells; **(b)** Collecting 11395 renal cells from the healthy mice, tSNE analysis indicated that AhR was also a few scattered within the S1-S3 segment of proximal tubule. MΦ, macrophage; IC-B, intercalated cell type B; IC-A, intercalated cell type A; CNT, connecting tubule; DCT, distal convoluted tubule; LH(AL), loop of Henle ascending loop; LH(DL), loop of Henle descending loop; PT(S3), S3 segment of proximal tubule; PT(S1, S2), S1,S2 segment of proximal tubule; EC, endothelial cell; MC, mesangial cell; Pod, podocyte; **(c)** UMAP displaying recategorized cell type names, and the expression level of AhR was obviously increase across PT cluster after ischemia reperfusion injury 4h. ATL, thin ascending limb of loop of Henle; CPC, principle cells of collecting duct in cortex; CTAL, thick ascending limb of loop of Henle in cortex; DTL, descending limb of loop of Henle; EC, endothelial cells; Fib, fibroblasts; ICA, type A intercalated cells of collecting duct; ICB, type B intercalated cells of collecting duct; MD, macula densa; MPC, principle cells of collecting duct in medulla; MTAL, thick ascending limb of loop of Henle in medulla; PEC, parietal epithelial cells; Per, pericytes; Uro, urothelium.


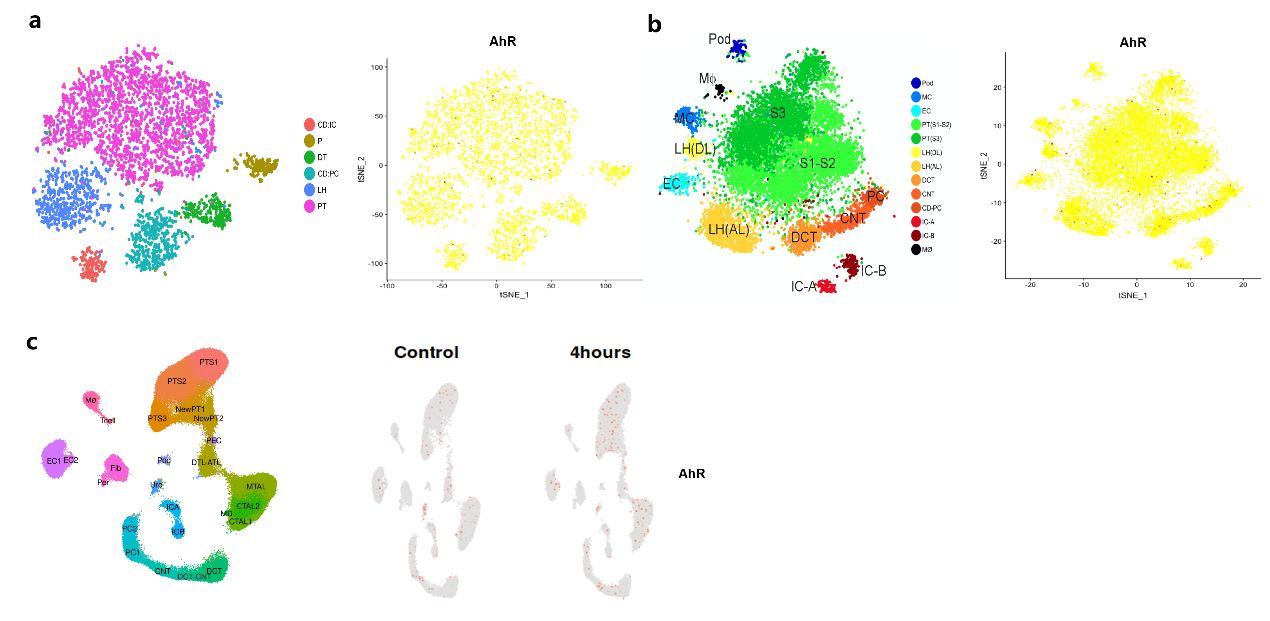


| Antibody | Firm | Country | Cat. |
| --- | --- | --- | --- |
| NGAL | Affinity | China | DF6816 |
| AhR | Affinity | China | AF6278 |
| Histone3 | Huabio | China | ET1701-64 |
| IL-6 | Huabio | China | EM170414 |
| p16 | Huabio | China | ER2001-30 |
| p53 | Huabio | China | EM1701-91 |
| TNF-α | Abcam | UK | Ab255275 |
| p21 | Abcam | UK | Ab109199 |
| EZH2 | Cell signaling Technology | USA | 5246s |
| IL-1β | Cell signaling Technology | USA | 31202s |
| H3K27me3 | Cell signaling Technology | USA | 9733s |

**Supplementary Table 1. Antibodies information used in the experiments.**

| Primer | Source | Forward (5’ - 3’) | Reverse (5’ - 3’) |
| --- | --- | --- | --- |
| GAPDH | mouse | GTCTTCACTACCATGGAGAAGG | TCATGGATGACCTTGGCCAG |
| TNF-α | mouse | GCGACGTGGAACTGGCAGAAG | GCCACAAGCAGGAATGAGAAGAGG |
| IL-6 | mouse | TAGTCCTTCCTACCCCAATTTCC | TAGTCCTTCCTACCCCAATTTCC |
| IL-1β | mouse | ACTGTGAAATGCCACCTTTTG | TGTTGATGTGCTGCTGTGAG |
| p16 | mouse | AACTCTTTCGGTCGTACCCC | GCGTGCTTGAGCTGAAGCTA |
| p21 | mouse | CCTGGTGATGTCCGACCTG | CCATGAGCGCATCGCAATC |
| p53 | mouse | TACTCCCCTGCCCTCAACAA | CATCGCTATCTGAGCAGCGC |
| EZH2 | mouse | AGTGACTTGGATTTTCCAGCAC | AATTCTGTTGTAAGGGCGACC |
| XO | mouse | GCTCTTCGTGAGCACACAGAAC | CCACCCATTCTTTTCACTCGGAC |
| Cat | mouse | CGGCACATGAATGGCTATGGATC | AAGCCTTCCTGCCTCTCCAACA |
| Sod1 | mouse | GGTGAACCAGTTGTGTTGTCAGG | ATGAGGTCCTGCACTGGTACAG |
| Sod2 | mouse | TAACGCGCAGATCATGCAGCTG | AGGCTGAAGAGCGACCTGAGTT |
| AhR | mouse | GCCGGTGCAGAAAACAGTAAA | GGTAACTGACGCTGAGCCTA |
| AhR-siRNA#1 | mouse | GAUCCUGGGAAACUCUACATT | UGUAGAGUUUCCCAGGAUCTT |
| AhR-siRNA#2 | mouse | GCACUGUCCUCUCAGAUUATT | UAAUCUGAGAGGACAGUGCTT |
| AhR-siRNA#3 | mouse | CCUUCCGCAAGAAGUAUAATT | UUAUACUUCUUGCGGAAGGTT |
| EZH2-siRNA#1 | mouse | GAGGAAGACUUCCGAAUAATT | UUAUUCGGAAGUCUUCCUCTT |
| EZH2-siRNA#2 | mouse | CGGUGUCAAACACCAAUAATT | UUAUUGGUGUUUGACACCGTT |
| EZH2-siRNA#3 | mouse | GCUCCUCUAACCAUGUUUATT | UAAACAUGGUUAGAGGAGCTT |

**Supplementary Table 2. The primers used in this study.**
