# Supplementary material for: Tubular aryl hydratocarbon receptor upregulates EZH2 to promote cellular senescence in cisplatin-induced acute kidney injury
Source: Cell Death Dis. 2023 Jan 12;14(1):18. doi: 10.1038/s41419-022-05492-3 (PMC9837170; doi:10.1038/s41419-022-05492-3)

Raw Western Blot Images

This file includes raw wester blot images presented in this paper. Notably, some of the images contain two or three bands, which are the result of the experiment being repeated. We chose the most appropriate bands to present in the manuscript, which has been marked with a red box. In addition, some original images have not been merged, so we upload them together.


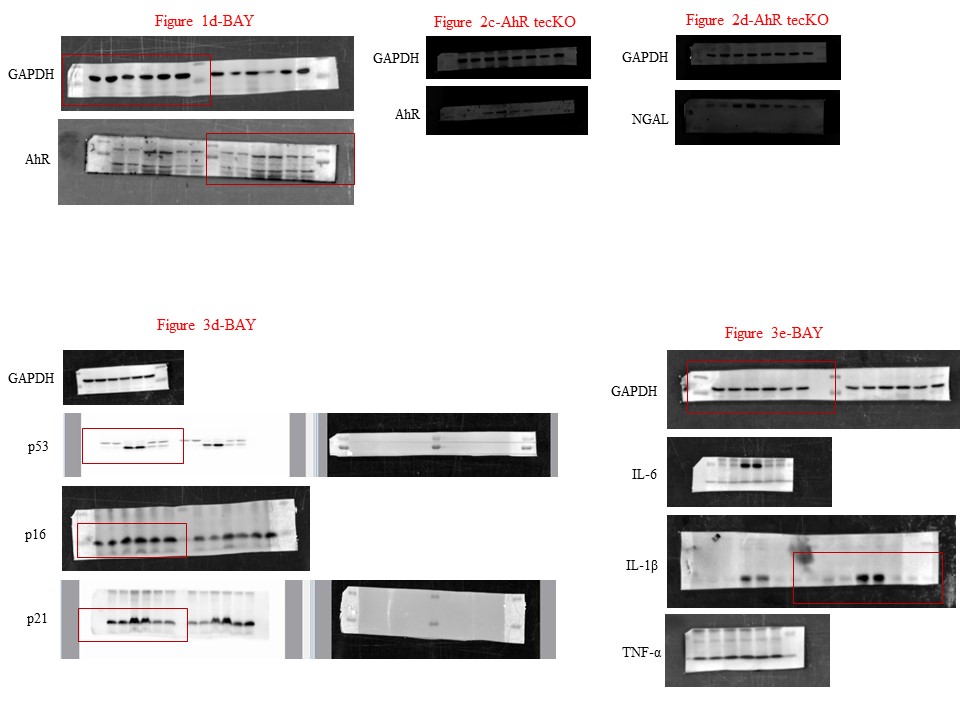


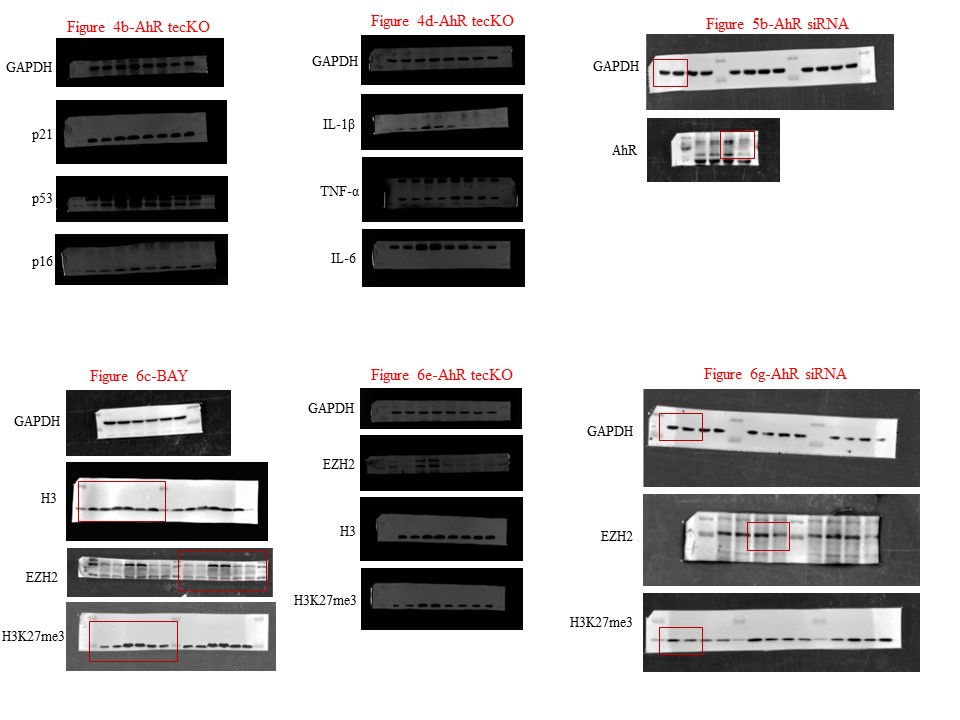


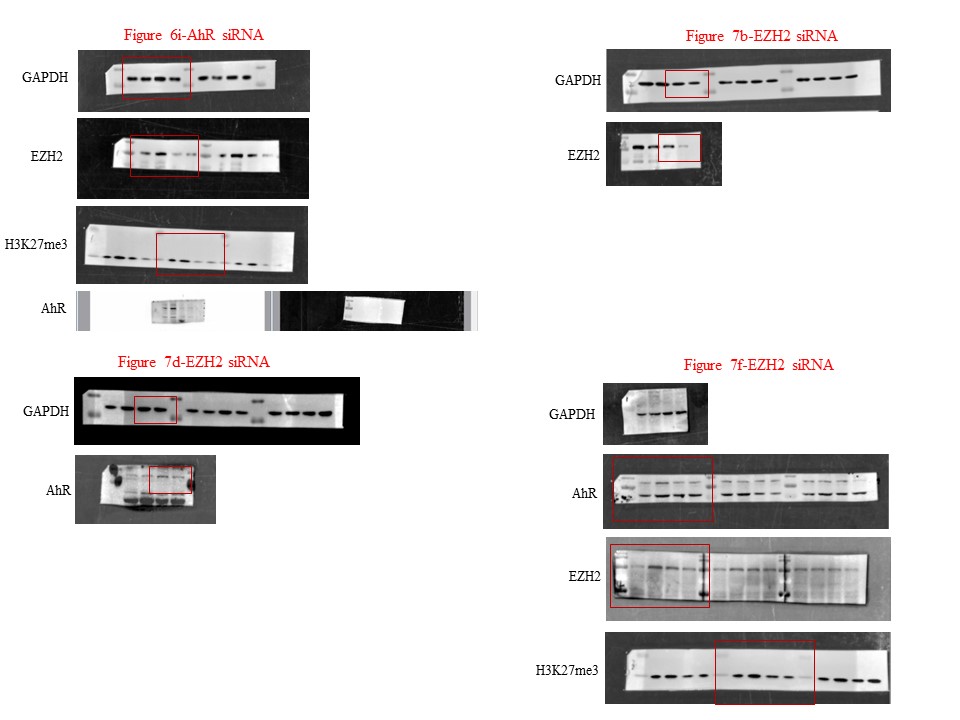

Supplement: Supplementary file 2 — Original Data File [file 41419_2022_5492_MOESM2_ESM.docx]
